# Tobacco use patterns and the social environment among university students in southern Ecuador

**DOI:** 10.3389/fpubh.2026.1904771

**Published:** 2026-08-07

**Authors:** Xavier Astudillo-Romero, Cristina Elisabeth Urgiles-Barahona, Patricio Espinosa Jaramillo

**Affiliations:** 1 Department of Health Sciences, Faculty of Health Sciences, Universidad Técnica Particular de Loja, Loja, Ecuador; 2 Faculty of Human Health, Universidad Nacional de Loja, Loja, Ecuador

**Keywords:** electronic nicotine delivery systems, peer influence, social environment, tobacco products, tobacco use, university students, vaping

## Abstract

**Background:**

Tobacco use remains a public health concern, particularly among young people, due to their greater vulnerability to smoking initiation and maintenance, as well as the emergence of new nicotine delivery devices that have diversified patterns of tobacco and nicotine use.

**Objective:**

This study aimed to describe tobacco use and identify the sociodemographic, family, and social factors associated with current tobacco use among university students in southern Ecuador.

**Methods:**

An observational, analytical, cross-sectional study was conducted in two universities in southern Ecuador. A non-probability convenience sampling approach was used. Of the 9,228 questionnaires collected, 9,091 complete records were included in the analysis. Descriptive analyses, chi-square tests, and multivariable binary logistic regression were performed, with adjusted odds ratios (aORs) and 95% confidence intervals (95% CIs) reported.

**Results:**

The mean age of the participants was 21.1 ± 2.9 years, and the mean age at smoking initiation among current tobacco users was 17.1 ± 2.3 years. Conventional cigarettes were the most frequently used tobacco product (96.5%), although 38.7% of participants reported using two or more tobacco products. Tobacco use was predominantly occasional, with only 6.0% reporting daily use. In the adjusted model, having a best friend who used tobacco (aOR = 3.08; 95% CI: 2.73–3.48), close friends who used tobacco (aOR = 2.34; 95% CI: 2.04–2.68), a sibling who used tobacco (aOR = 1.44; 95% CI: 1.26–1.64), a romantic partner who used tobacco (aOR = 1.37; 95% CI: 1.19–1.58), and classmates who used tobacco (aOR = 1.35; 95% CI: 1.21–1.50) were independently associated with current tobacco use. Female sex (aOR = 0.34; 95% CI: 0.31–0.38) and being exclusively engaged in academic studies (aOR = 0.81; 95% CI: 0.71–0.91) were associated with a lower likelihood of current tobacco use.

**Conclusion:**

Tobacco use among university students was primarily associated with smoking behaviors within peer networks, particularly those of best friends and close friends. These findings support the need for university-based prevention strategies targeting the social environment, group norms, and peer influence.

## 1 Introduction

Tobacco use remains one of the leading preventable causes of disease, disability, and premature death worldwide. Although global trends indicate a decline in tobacco use prevalence, it continues to represent a major public health problem in low- and middle-income countries, where a substantial proportion of tobacco users reside. Young adults remain particularly vulnerable to tobacco use due to sociocultural pressures, tobacco industry marketing strategies, and the expanding market of emerging nicotine products, including electronic cigarettes, heated tobacco products, and nicotine pouches, among others, which are reshaping traditional patterns of tobacco use. Their increasing availability and social acceptance have contributed both to the continuation of tobacco use among existing users and to the recruitment of new users. This heterogeneity in tobacco use patterns underscores the need to strengthen tobacco control policies and smoking cessation programs tailored to the sociocultural context, as well as to generate evidence that clarifies the public health implications of these emerging products (1, 2).

The university years coincide with a critical stage of development characterized by increasing personal autonomy, academic pressure, exposure to new social networks, and access to tobacco and nicotine products. Within this context, engagement in risk behaviors may begin, intensify, or become established, particularly during the early years of higher education. Tobacco use is one of these behaviors and may become more prevalent during this period, persist over time, or evolve into other forms of nicotine consumption. Understanding the characteristics of tobacco use among university students is particularly important for designing timely, targeted, and context-specific preventive interventions (3, 4).

International evidence shows substantial variability in the prevalence of tobacco use among university students, with estimates ranging from 8 to 35%, depending on the sociocultural context, local regulations, type of institution, and characteristics of the study population (5). Beyond this variability, recent studies consistently indicate that tobacco use among university students is influenced not only by individual characteristics but also by environmental factors. Reported associated factors include sex, academic stress, exposure to secondhand smoke, the presence of family members who use tobacco, and peer influence. These findings suggest that preventive strategies targeting university students should address not only individual determinants but also family, social, and institutional factors (6).

Patterns of tobacco use have also diversified. Although conventional cigarettes remain the predominant form of tobacco use in many settings, the use of alternative products such as electronic cigarettes, vaping devices, and heated tobacco products has steadily increased, sometimes in combination with conventional cigarettes. This coexistence of products may increase nicotine exposure, reinforce nicotine dependence, and hinder smoking cessation attempts. Among university students, recent studies have described combined patterns of smoking and vaping associated with individual factors, favorable attitudes toward tobacco use, social influence, product availability, and situational triggers. In this context, peer pressure, socialization, academic stress, family environment, and employment status have emerged as important factors for understanding tobacco and nicotine use among university students. These findings reinforce the need to implement interventions that target not only individual behaviors but also social norms, product accessibility, and risk perception related to tobacco and nicotine products (7–10).

Despite the growing body of international evidence, information on tobacco and nicotine use among university students in Latin America remains limited and heterogeneous. In Ecuador, studies addressing this issue are scarce and have focused primarily on estimating prevalence, with less evidence available regarding patterns of use of emerging tobacco products, exposure to secondhand smoke, and factors associated with tobacco use among university students. Updated information on these dimensions is essential to inform institutional policies and evidence-based prevention strategies. Therefore, the present study aimed to describe tobacco and related product use and to identify the sociodemographic, family, and social factors associated with current tobacco use among university students in southern Ecuador.

## 2 Materials and methods

An observational, analytical, cross-sectional study was conducted as part of a research project carried out at two universities in southern Ecuador, one public and one private. The source population consisted of undergraduate students enrolled in on-campus academic programs. A non-probability convenience sampling approach was used. The questionnaire link was disseminated through institutional channels to the eligible student population at both universities, and all students who voluntarily agreed to participate, provided informed consent, and completed the questionnaire during the data collection period were included. Eligible participants were first-year university students, aged 18 years or older, who were able to provide informed consent. Of the 9,228 questionnaires collected, 137 records were excluded because of incomplete information in one or more variables required for the analyses. Consequently, the final analyses were performed using a complete-case analysis, including 9,091 students.

Data were collected using a structured self-administered questionnaire delivered online through Microsoft Forms. The questionnaire included sociodemographic, academic, family, social, and tobacco- and nicotine-related variables. In addition, risk perception was assessed using as a reference the instrument described in the report developed by the Centro de Estudios Socio-Culturales de Chile on health risk perception associated with cigarette displays at points of sale (11). For the present study, the instrument underwent face and content validity assessment by a panel of five experts, yielding an Aiken’s V coefficient of 0.98.

Statistical analyses were performed using IBM SPSS Statistics, version 29. Quantitative variables were summarized using measures of central tendency and dispersion, whereas qualitative variables were described using absolute frequencies, percentages, and 95% confidence intervals (95% CIs). Associations between the independent variables and current tobacco use were assessed using the chi-square test and crude odds ratios (ORs) with their corresponding 95% confidence intervals. Subsequently, a multivariable binary logistic regression model was fitted using the Enter method, simultaneously including the sociodemographic, family, and social variables considered in the study to estimate their independent associations with current tobacco use. Adjusted odds ratios (aORs) and their corresponding 95% confidence intervals (95% CIs) were estimated.

Variables related to tobacco consumption intensity (average number of tobacco products consumed per day and per week) were collected as grouped ordinal categories (1, 2–10, 11–20, and ≥21 products). Therefore, count-data regression models, such as Poisson or negative binomial regression, were not considered appropriate. These variables were analyzed descriptively according to the study objectives. Multicollinearity among the family and social environment predictors included in the multivariable model was assessed using variance inflation factors (VIFs) and tolerance values. Model fit was evaluated using the Hosmer–Lemeshow goodness-of-fit test. A two-sided *p*-value < 0.05 was considered statistically significant. The study was approved by the Research Ethics Committee for Human Subjects of the University of Cuenca under approval letter CEISH-2023-012EO-IE.

## 3 Results

### 3.1 Sociodemographic characteristics of participants

The study population comprised 9,091 university students, of whom 2,608 reported current tobacco use, corresponding to a prevalence of 28.7% (95% CI: 27.8–29.6). The mean age was 21.08 ± 2.92 years. Most participants were female (57.36%), single (95.86%), lived with their family of origin (60.99%), and attended the public university (77.33%). Among current tobacco users, males (63.77%) and students who combined study and work (28.91%) predominated. Detailed information is presented in Table 1.

**Table 1:** Sociodemographic characteristics of the study population according to tobacco use status (smoker vs. non-smoker).

Variable	Total population		Non-smokers		Smokers		*p* value
Age	Mean	SD	Mean	SD	Mean	SD	
	21.08 (21)	2.92 (19–22)	21.23 (21)	2.90 (19–21)	21.02 (20)	2.93 (19–22)	0.002*
Minimum	16		16		16		
Maximum	56		56		43		
	*N*	%	*N*	%	*N*	%	
Gender							
Male	3,859	42.45%	2,196	33.87%	1,663	63.77%	< 0.001**
Female	5,215	57.36%	4,277	65.97%	938	35.97%	
Other	17	0.19%	10	0.15%	7	0.27%	
Ethnic group							
Mestizo	8,759	96.35%	6,234	96.16%	2,525	96.82%	0.157**
Afro-Ecuadorian	46	0.51%	32	0.49%	14	0.54%	
Indígenus	143	1.57%	114	1.76%	29	1.11%	
Montubio	23	0.25%	19	0.29%	4	0.15%	
White	120	1.32%	84	1.30%	36	1.38%	
Occupation							
Student	6,992	76.91%	5,138	79.25%	1854	71.09%	< 0.001***
Student and employed	2099	23.09%	1,345	20.75%	754	28.91%	
Marital status							
Single	8,715	95.86%	6,222	95.97%	2,493	95.59%	0.316
Married	177	1.95%	129	1.99%	48	1.84%	
Divorced	37	0.41%	22	0.34%	15	0.58%	
Widowed	11	0.12%	6	0.09%	5	0.19%	
Cohabiting/Common-law union	151	1.66%	104	1.60%	47	1.80%	
Living arrangement							
Living alone	1757	19.33%	1,247	19.23%	510	19.56%	0.378
Living with family of origin	5,545	60.99%	3,947	60.88%	1,598	61.27%	
Living with other relatives	1,287	14.16%	926	14.28%	361	13.84%	
Living with friends/roommates	215	2.36%	146	2.25%	69	2.65%	
Living with partner	287	3.16%	217	3.35%	70	2.68%	
University type							
Private	2061	22.67%	1,395	21.52%	666	25.54%	<0.001***
Públic	7,030	77.33%	5,088	78.48%	1942	74.46%	

SD, standard deviation; *t-test; ** χ^2^ test; *** Fisher’s exact test.

### 3.2 Characteristics of tobacco and nicotine use

Among current tobacco users, conventional cigarettes were the most commonly used tobacco product (96.47%). Use of nicotine-containing vaping devices (22.51%), electronic cigarettes (21.28%), nicotine-free vaping devices (14.21%), and water pipes (shisha) (3.76%) was also reported. The most common places for tobacco use were outdoor public spaces (62.85%), indoor public spaces (47.78%), and university campuses (30.02%). The predominant purchasing pattern was buying cigarettes individually (84.43%), mainly from supermarkets or shopping centers (59.28%), bars, nightclubs, or entertainment venues (49.35%), and locations surrounding university campuses (30.87%). Tobacco use was primarily financed through participants’ own employment (45.86%) or monthly financial support from their parents (40.03%). Detailed information is presented in Table 2.

**Table 2:** Tobacco consumption characteristics: product type, place of use, and acquisition patterns.

	Frecuency *n*	Percentage (%)
Type of tobacco product consumed*		
Conventional cigarettes	2,511	96.47
Electronic cigarettes	554	21.28
Nicotine-containing vape	586	22.51
Nicotine-free vape	370	14.21
Smokeless tobacco (powdered tobacco)	27	1.04
Waterpipe (shisha)	98	3.76
Other products	8	0.31
Tobacco product purchased*		
Single cigarettes	2,202	84.43
Pack of 10 cigarettes	562	21.55
Pack of 20 cigarettes	218	8.36
Electronic cigarettes	178	6.83
Nicotine-containing vape	295	11.31
Nicotine-free vape	166	6.37
Other products	14	0.54
Source of money used to purchase tobacco*		
Own employment/income	1,196	45.86
Friends or relatives	364	13.96
Others	87	3.34
Allowance received from parents	1,044	40.03
Pooling money with friends	443	16.99
Place of tobacco use*		
At home	531	20.36
University campus	783	30.02
Outdoor public places	1,639	62.85
Indoor public places	1,246	47.78
In a vehicle	271	10.39
Other locations	31	1.19
Place of tobacco purchase*		
On campus	265	10.16
Around campus	805	30.87
Supermarket or shopping mall	1,546	59.28
Bars, nightclubs, and entertainment venues	1,287	49.35
Online purchase	80	3.07

^*^Percentages may exceed 100% because participants were allowed to select more than one response option.

### 3.3 Frequency, intensity, and temporal pattern of tobacco use

The mean age at tobacco use initiation was 17.05 ± 2.32 years. Regarding frequency of use, 48.27% reported using tobacco once every two months or less frequently, 27.68% at least once per month, 18.02% at least once per week, and 6.02% daily. Regarding the longest uninterrupted period of tobacco use, 60.31% reported continuous use for one to three weeks, whereas 4.49% indicated that they had never stopped smoking since initiating tobacco use. Concerning the most recent tobacco use episode, 18.17% had smoked within the previous 24 h. Most participants reported not experiencing a strong desire to smoke again (62.27%). On a typical day, 80.52% reported consuming only one tobacco product, whereas 18.37% consumed between two and ten products. Similarly, during a typical week, 66.64% reported consuming one tobacco product and 28.11% consumed between two and ten products. In addition, 61.3% reported using a single type of tobacco product, whereas 38.7% used two or more different types of tobacco products. Detailed information is presented in Table 3.

**Table 3:** Frequency, intensity, and temporal patterns of tobacco use.

Variable		
Age at smoking initiation (years)	Mean ± SD	Min/Max
	17.05 ± 2.32	8–26
What is the longest period you have smoked continuously?	*N*	%
One to three weeks	1,573	60.31
One month	329	12.62
Three months	202	7.75
Six months	160	6.13
One year	86	3.3
More than one year	141	5.41
I have not stopped smoking since I started	117	4.49
When did you last use tobacco?		
Within the last 24 h	474	18.17
More than one day ago	393	15.07
More than one week ago	734	28.14
More than one month ago	602	23.08
More than three months ago	405	15.53
After smoking a cigarette, how long does it take before you feel the urge to smoke again?		
Immediately	35	1.34
Less than one hour	76	2.91
Less than 12 h	111	4.26
Less than one day	147	5.64
Less than one week	245	9.39
Less than one month	154	5.9
More than one month	216	8.28
I never feel a strong urge to smoke again	1,624	62.27
Frequency of tobacco use		
Once every two months or less frequently	1,259	48.27
At least once per month	722	27.68
At least once per week	470	18.02
Daily	157	6.02
How many tobacco products do you consume in an average week?		
One	2,100	80.52
2–10	479	18.37
11–20	21	0.81
21 or more	8	0.31
How many tobacco products do you consume on an average day?		
One	1738	66.64
2–10	733	28.11
11–20	95	3.64
21 or more	42	1.61
Different tobacco products consumed	*N*	% (IC 95%)
Only one type of product	1,599	61.3 (59.4–63.2)
Up to three different types of products	868	33.3 (31.5–35.1)
Four or more different types of products	141	5.4 (4.6–6.3)

### 3.4 Tobacco use in the social and family environment

Tobacco use was more frequently reported within the social environment than within the family environment. In the overall study population, the highest prevalence of tobacco use was reported among close friends (63.93%), classmates (40.35%), and best friends (35.47%). Within the family environment, tobacco use was most frequently reported among fathers and/or mothers (31.72%) and siblings (25.07%). Among current tobacco users, 85.89% reported having close friends who used tobacco, and 65.41% indicated that their best friend used tobacco. The category “no one uses tobacco” was more frequently reported by non-users (8.08%) than by current tobacco users (2.38%). Detailed information is presented in Table 4.

**Table 4:** Tobacco use among individuals in the social environment according to participants’ smoking status.

Person in the social circle who uses tobacco	Total population		Non-smokers		Smokers	
	Frecuency *n*	Percentage (%)	Frecuency *n*	Percentage (%)	Frecuency *n*	Percentage (%)
Father and/or mother	2,884	31.72	1786	27.55	1,098	42.10
Brother and/or sister	2,279	25.07	1,240	19.13	1,039	39.84
Grandfather and/or grandmother	1,597	17.57	916	14.13	681	26.11
Other cohabiting relative	1893	20.82	1,031	15.90	862	33.05
Other person living in the household	1,563	17.19	818	12.62	745	28.57
Household guests	3,334	36.67	2035	31.39	1,299	49.81
Close friends	5,812	63.93	3,572	55.10	2,240	85.89
Best friend	3,225	35.47	1,519	23.43	1706	65.41
Romantic partner	1914	21.05	997	15.38	917	35.16
Classmates	3,668	40.35	2,377	36.67	1,291	49.50
Nobody	586	6.45	524	8.08	62	2.38
Others	2,695	29.64	1,643	25.34	1,052	40.34

### 3.5 Perceived tobacco use in the social environment

The perceived influence of tobacco use by family members and friends on participants’ own tobacco use differed significantly according to participants’ tobacco use status (*p* < 0.001). These findings suggest an association between perceived social influence and tobacco use among university students; however, no causal direction can be inferred because of the cross-sectional design of the study. Detailed information is presented in Table 5.

**Table 5:** Perception of one’s own tobacco use relative to tobacco use among people in the social environment.

	Frecuency *n*	Percentage (%)	Frecuency *n*	Percentage (%)	Frecuency *n*	Percentage (%)	Frecuency *n*	Percentage (%)	Frecuency *n*	Percentage (%)
Self-perceived tobacco use compared with tobacco use among people in the social environment										
	Father/Mother		Household guests		Sibling		Close friends		Grandparent	
High	210	8.05	119	4.56	104	3.99	439	16.83	136	5.21
Moderate	282	10.81	415	15.91	301	11.54	927	35.54	137	5.25
Low	606	23.24	728	27.91	566	21.70	863	33.09	377	14.46
Does not smoke	1,510	57.90			1,637	62.77	379	14.53	1958	75.08
	Best friend		Other relative living with me		Partner		Other person living with me		Classmates	
High	312	11.96	116	4.45	137	5.25	95	3.64	165	6.33
Moderate	594	22.78	229	8.78	231	8.86	202	7.75	349	13.38
Low	760	29.14	473	18.14	525	20.13	427	16.37	531	20.36
Does not smoke	942	36.12	1790	68.63	1715	65.76	1884	72.24	1,563	59.93
Perception of tobacco use by cohabitants										
	Father/Mother		Grandparent		Grandparent		Other relative living with me		Partner	
High	158	6.06	103	3.95	95	3.64	80	3.07	115	4.41
Moderate	251	9.62	272	10.43	138	5.29	170	6.52	239	9.16
Low	426	16.33	507	19.44	320	12.27	430	16.49	445	17.06
Does not smoke	1773	67.98	1726	66.18	2055	78.80	1928	73.93	1809	69.36
	Other person living with me		Household guests		Best friend		Close friends		Others	
High	60	2.30	113	4.33	316	12.12	479	18.37	151	5.79
Moderate	163	6.25	368	14.11	544	20.86	864	33.13	294	11.27
Low	409	15.68	658	25.23	670	25.69	721	27.65	453	17.37
Does not smoke	1976	75.77	1,469	56.33	1,078	41.33	544	20.86	1710	65.57

### 3.6 Perceived influence of the social environment

The perceived influence of tobacco use by family members and friends on participants’ own tobacco use differed significantly according to participants’ tobacco use status (*p* < 0.001). These findings suggest an association between perceived social influence and tobacco use among university students; however, no causal direction can be inferred because of the cross-sectional design of the study. Detailed information is presented in Table 6.

**Table 6:** Perceived influence of family and social environment on current tobacco use.

Variable assessed		Co-residing relatives				Close friends			
		Tobacco use status				Tobacco use status			
		Yes	No	Test	*p*-value	Yes	No	Test	*p*-value
Their tobacco use influences me to smoke	Yes	28.53%	2.51%	1401.247	<0.001	21.43%	32.73%	114.162	<0.001
	No	71.47%	97.49%			78.57%	67.27%		
Their tobacco use influences me not to smoke	Yes	21.59%	31.30%	86.027	<0.001	25.77%	60.88%	917.575	<0.001
	No	78.41%	68.70%			74.23%	39.12%		
Their non-use of tobacco influences me not to smoke	Yes	49.88%	66.19%	208.808	<0.001	52.80%	6.39%	208.808	<0.001
	No	50.12%	33.81%			47.20%	93.61%		

### 3.7 Sociodemographic, family-related, and social factors associated with current tobacco use

A multivariable logistic regression model adjusted for sociodemographic, family, and social variables was constructed. The model was globally significant (χ^2^ = 2146.14; *p* < 0.001), yielded a Nagelkerke pseudo-R^2^ of 0.302, indicating an improvement in model fit compared with the null model, and correctly classified 77.1% of cases. Although the Hosmer–Lemeshow goodness-of-fit test was statistically significant (χ^2^ = 34.34; df = 8; *p* < 0.001), this result should be interpreted with caution because of the well-recognized sensitivity of this test in large samples. No evidence of problematic multicollinearity was identified among the predictors included in the model (VIF: 1.04–2.69; tolerance: 0.372–0.959).

The strongest associations were observed for variables related to the social environment. Students whose best friend used tobacco had approximately three times higher odds of current tobacco use (aOR = 3.08; *p* < 0.001), followed by those close friends who used tobacco (aOR = 2.34; *p* < 0.001). Significant associations were also identified for having siblings who used tobacco (aOR = 1.44; *p* < 0.001), a romantic partner who used tobacco (aOR = 1.37; *p* < 0.001), and classmates who used tobacco (aOR = 1.35; *p* < 0.001). Among the sociodemographic variables, female students had lower odds of current tobacco use than males (aOR = 0.34; *p* < 0.001), whereas students who were exclusively engaged in academic studies had lower odds of current tobacco use than those who combined study and work (aOR = 0.81; *p* < 0.001). In contrast, parental tobacco use and university attendance were not independently associated with current tobacco use. Detailed information is presented in Table 7.

**Table 7:** Multivariable logistic regression model of factors associated with current tobacco use among university students.

Variable	B	Standard error	Adjusted OR (Exp(B))	95% CI	*p*-value
Female sex (vs. male)	−1.08	0.055	0.339	0.305–0.378	<0.001
Only studying (vs. studying and working)	−0.22	0.062	0.805	0.711–0.906	<0.001
Public university (vs. private)	0.008	0.062	1.008	0.893–1.138	0.893
Father and/or mother uses tobacco	0.075	0.064	1.078	0.951–1.222	0.239
Sibling uses tobacco	0.364	0.067	1.439	1.262–1.641	<0.001
Romantic partner uses tobacco	0.315	0.074	1.37	1.185–1.584	<0.001
Close friends use tobacco	0.849	0.07	2.338	2.038–2.681	<0.001
Best friend uses tobacco	1.125	0.062	3.08	2.728–3.478	<0.001
Classmates use tobacco	0.298	0.054	1.347	1.212–1.498	<0.001

Model fit statistics: χ^2^ = 2146.14, *p* < 0.001; Cox and Snell R^2^ = 0.211; Nagelkerke R^2^ = 0.302; overall classification accuracy = 77.1%.

## 4 Discussion

Tobacco use is a socially tolerated public health problem, and its impact therefore extends beyond the clinical setting. Among university students, this behavior occurs during a life stage characterized by changes in autonomy, the construction of social identity, and environmental influence, making the adoption or consolidation of risk behaviors a common phenomenon. Tobacco use is one such behavior and, when initiated or reinforced during this stage, tends to persist into adulthood, increasing the future burden of disease. The present study provides a comprehensive overview of the characteristics associated with tobacco use among university students in southern Ecuador by describing users and their environment, while also analyzing perceptions of personal and environmental tobacco use in order to identify factors that may motivate or influence its initiation.

Regarding the prevalence of current tobacco use, estimates in Latin America range from 15 to 43% (12–17), although few studies report this indicator. Outside the Latin American context, the prevalence of current tobacco use ranges from 7 to 52% (6, 7, 18–21). In the present study, 28.7% of university students reported current tobacco use, with a lower proportion among women, suggesting a pattern comparable to that observed in other contexts (21–24).

In describing tobacco users, the findings show similarities with international studies. The age at initiation of tobacco use in our population (17.05 ± 2.32 years) is consistent with that reported by Brożek et al., Prigitano et al., and Bolshakova et al., in which initiation occurs around 16 years of age (15 to 20 years) (19, 22, 23). The most frequently used product was the conventional cigarette, in agreement with findings reported in European studies (22, 23).

Regarding the diversity of products used, 38.7% of users reported using two or more different tobacco or nicotine products. This proportion is higher than that reported by Ahmed et al., who described approximately 30% poly-use in a university population (7), and considerably higher than that observed by Pokhaznikova et al., who reported a frequency of 6.8% (21). These differences may be related to the characteristics of the populations studied, the availability of emerging products, and the different operational definitions used to characterize poly-use.

Regarding places of use, the present study reports greater tobacco use in public places and on university campuses, similar to that reported by Kaplan et al. (25). Tobacco acquisition occurs mainly in supermarkets, shopping centers, bars, and other leisure spaces, with areas surrounding university campuses also representing an important point of access. These findings suggest that the physical availability of tobacco products remains a relevant factor for use among university students (26, 27).

Perceived tobacco use differed according to the type of social relationship. Students perceived higher levels of tobacco use among close friends and best friends than among family members, similar to that reported by Bin Abdulrahman et al. and Pokhaznikova et al. (21, 24). This finding is relevant because it suggests that social norms related to tobacco use may be constructed mainly within closer peer groups rather than within the immediate family environment. Greater visibility of tobacco use among friends may favor processes of normalization and social acceptance of the habit during the university stage. In addition, it was interesting to observe that smokers perceived their own use as low or nonexistent when compared with the tobacco use of people in their family environment; however, when compared with their friends, they perceived it as moderate or high.

Regarding factors associated with current tobacco use, the multivariable analysis showed that the strongest associations were found in the immediate social environment. Having a best friend who used tobacco and close friends who used tobacco were the variables with the greatest magnitude of association, even after controlling for sociodemographic and family variables. Tobacco use by siblings, romantic partners, and classmates also remained associated. These results are consistent with previous studies identifying peer influence as one of the most important determinants of tobacco use among university students (24, 28–30). In contrast, parental tobacco use lost statistical significance after multivariable adjustment, suggesting that during the university stage peer influence may carry greater weight than family influence on tobacco-use behavior.

These findings can be interpreted in light of Bandura’s Social Learning Theory and models of peer influence. From this perspective, tobacco use does not depend exclusively on individual characteristics, but also on processes of observation, modeling, imitation, social reinforcement, and normalization within the reference group. The greater magnitude of association observed for best friends and close friends suggests that university prevention interventions should incorporate components targeting social norms, group dynamics, and peer networks (29, 31).

The perceived influence of family members and friends also differed significantly according to students’ tobacco-use status. Although the cross-sectional design precludes establishing causal relationships, these results suggest that perceived acceptance or social influence may play a relevant role in university tobacco-use behavior. This finding is consistent with literature highlighting the role of perceived social norms, peer pressure, and socialization processes in the initiation and maintenance of tobacco use among young adults (10, 12).

An additional limitation of the study is that participants were clustered within universities and faculties, which could introduce some degree of intra-institutional correlation. In response to this possibility, the association between faculty and current tobacco use was explored, showing a statistically significant association; however, the effect size was small (Cramér’s V = 0.135). Therefore, the application of a multilevel model was not considered justified for addressing the main objective of the study, which focused on individual, family, and social factors associated with tobacco use. Nevertheless, future studies specifically aimed at evaluating contextual effects could benefit from hierarchical models that estimate the variability attributable to universities or faculties. In addition, although the Hosmer–Lemeshow test was statistically significant, this result should be interpreted with caution because of the high sensitivity of this test in large samples.

Finally, these results reflect the importance of developing preventive interventions directed not only at the individual but also at their immediate social environment. The observed influence of best friends, close friends, and other members of the social network suggests that tobacco-use prevention and cessation strategies in the university setting may achieve better results if they incorporate peer-network-based approaches, the promotion of healthy social norms, and the strengthening of protective factors within the student community. In addition, the coexistence of conventional cigarettes and new nicotine products highlights the need for institutional policies and health promotion programs to comprehensively address all forms of tobacco and nicotine use.

## 5 Conclusion

Current tobacco use among university students in southern Ecuador showed a relevant prevalence, with initiation occurring during late adolescence, predominance of conventional cigarette use, and the presence of poly-use of tobacco and nicotine products. Although tobacco use was predominantly occasional, the coexistence of traditional and emerging products reflects a heterogeneous pattern of use that requires continued surveillance and institutional intervention.

The factors most strongly associated with current tobacco use were tobacco-use behaviors within the immediate social environment, particularly among best friends, close friends, romantic partners, classmates, and siblings. These findings highlight the importance of addressing tobacco use from a social perspective rather than focusing exclusively on individual-level factors.

The findings suggest the need to implement comprehensive prevention strategies centered on peer networks, the promotion of healthy social norms, reducing the availability of tobacco and nicotine products, and strengthening risk perception among university students, including before they enter higher education.

## Data Availability

The datasets supporting the conclusions of this article will be made available by the authors upon reasonable request. Any shared dataset will be fully anonymized to protect participants’ privacy and confidentiality.
